# Patient and relative experiences of the ReSPECT process in the community: an interview-based study

**DOI:** 10.1186/s12875-024-02283-x

**Published:** 2024-04-17

**Authors:** Karin Eli, Jenny Harlock, Caroline J. Huxley, Celia Bernstein, Claire Mann, Rachel Spencer, Frances Griffiths, Anne-Marie Slowther

**Affiliations:** Warwick Medical School, University of Warwick, Gibbet Hill Campus, Coventry, CV4 7AL UK

**Keywords:** Emergency care and treatment planning (ECTP), The recommended Summary Plan for Emergency Care and Treatment (ReSPECT), Qualitative research, Patient and relative experiences

## Abstract

**Background:**

The Recommended Summary Plan for Emergency Care and Treatment (ReSPECT) was launched in the UK in 2016. ReSPECT is designed to facilitate meaningful discussions between healthcare professionals, patients, and their relatives about preferences for treatment in future emergencies; however, no study has investigated patients’ and relatives’ experiences of ReSPECT in the community.

**Objectives:**

To explore how patients and relatives in community settings experience the ReSPECT process and engage with the completed form.

**Methods:**

Patients who had a ReSPECT form were identified through general practice surgeries in three areas in England; either patients or their relatives (where patients lacked capacity) were recruited. Semi-structured interviews were conducted, focusing on the participants’ understandings and experiences of the ReSPECT process and form. Data were analysed using inductive thematic analysis.

**Results:**

Thirteen interviews took place (six with patients, four with relatives, three with patient and relative pairs). Four themes were developed: (1) ReSPECT records a patient’s wishes, but is entangled in wider relationships; (2) healthcare professionals’ framings of ReSPECT influence patients’ and relatives’ experiences; (3) patients and relatives perceive ReSPECT as a do-not-resuscitate or end-of-life form; (4) patients’ and relatives’ relationships with the ReSPECT form as a material object vary widely. Patients valued the opportunity to express their wishes and conceptualised ReSPECT as a process of caring for themselves and for their family members’ emotional wellbeing. Participants who described their ReSPECT experiences positively said healthcare professionals clearly explained the ReSPECT process and form, allocated sufficient time for an open discussion of patients’ preferences, and provided empathetic explanations of treatment recommendations. In cases where participants said healthcare professionals did not provide clear explanations or did not engage them in a conversation, experiences ranged from confusion about the form and how it would be used to lingering feelings of worry, upset, or being burdened with responsibility.

**Conclusions:**

When ReSPECT conversations involved an open discussion of patients’ preferences, clear information about the ReSPECT process, and empathetic explanations of treatment recommendations, working with a healthcare professional to co-develop a record of treatment preferences and recommendations could be an empowering experience, providing patients and relatives with peace of mind.

**Supplementary Information:**

The online version contains supplementary material available at 10.1186/s12875-024-02283-x.

## Introduction

The Recommended Summary Plan for Emergency Care and Treatment (ReSPECT) is an emergency care and treatment planning (ECTP) process launched in the UK in 2016. Key to the ReSPECT process is the facilitation of meaningful discussions between healthcare professionals, patients and their relatives about preferences for treatment in future emergencies, including cardiopulmonary resuscitation (CPR) [1]. This process is accompanied by the ReSPECT form, which records a summary of the discussion, including treatment recommendations, to be signed by the healthcare professional and held by the patient [1]. While a patient’s preferences, wishes and values inform the recommendations recorded on the ReSPECT form, the recommendations reflect shared decision-making rather than directly setting out the patient’s wishes [2].

We have recently completed a programme of work, researching the ReSPECT process in acute hospitals in England, which has produced several qualitative studies of clinician and patient experiences, exploring the ReSPECT process from different angles. An observation- and interview-based study of ReSPECT conversations in hospital found that conversations could be exploratory or persuasive, with doctors taking varying stances toward the extent to which patients’ and relatives’ preferences should direct the recommendations [3]. An ethnographic study found that some ReSPECT conversations in hospital were not performed due to time constraints and the sensitivity of timing these conversations, while another study found that, in hospital settings, mismatches between doctors’ and patients’ priorities and understandings led to incomplete ReSPECT conversations [4, 5]. In addition, interview and focus group studies found that both hospital-based and primary care doctors viewed ReSPECT as a process that required good rapport and careful negotiation of patients’ and relatives’ emotions [6, 7]. Finally, an interview study found that, during an acute admission, patients and relatives tended to feel unprepared for ReSPECT conversations and confused about how ReSPECT may affect their future care [8].

Despite the centrality of patients and relatives to the ReSPECT process, no study so far has investigated patients’ and relatives’ experiences of ReSPECT in the community. In addition to our earlier programme of work, we have identified only one other qualitative study on the ReSPECT process; this study examined how general practitioners (GPs) and social care staff experienced ReSPECT in the care home context [9]. Moreover, a recent qualitative systematic review found only one study that focused on patient experiences of CPR-related conversations in the UK, predating the ReSPECT process [10].

The current study explores how the ReSPECT process and form are understood, perceived, and experienced by patients and their relatives in community contexts [11]. This exploration is part of a larger study on the use of ReSPECT in primary and community care.

## Methods

### Participant recruitment

Patients and relatives were recruited through 13 GP practices, representing three geographic areas in England. Based on Index of Multiple Deprivation (IMD) deciles [12], Area 1 included GP practices across a wide socioeconomic range (IMD deciles 1 to 8), Area 3 included GP practices from areas with high levels of relative deprivation (IMD deciles 3 and 4), and Area 2 included GP practices from areas with the highest levels of relative deprivation (IMD deciles 1 and 2).

Participating GP practices identified patients who had a ReSPECT form completed in the last 12 months and contacted eligible patients or the next of kin of eligible patients who were identified as not having capacity. Patients (on the national data opt out register) who had previously opted out of their data being used for purposes other than clinical care were excluded. The study also excluded patients identified as being in hospital or in the final stages of a terminal illness. The GP practices sent potential participants an invitation letter and a brief information sheet about the study by post. Those who were interested in participating sent back an expression of interest to the study team. A researcher then contacted these potential participants by telephone to provide more information about the study. If participants continued to express interest in the study, they were sent an information sheet and consent form either by post or by email, and an interview was arranged. Participants could choose whether to have the interview in person or by telephone.

The study was approved by the London South East NHS Research Ethics Committee (REC 21/LO/0455). The participants provided informed consent before the interviews, either in writing (in-person interviews) or verbally (telephone interviews). All participants have been given pseudonyms and identifying details have been concealed or altered in this manuscript.

### Data collection

The study team developed two interview topic guides, one for patient participants and one for relative participants. The topic guides were designed to capture key elements in the ReSPECT process experience, including the ReSPECT conversation and the feelings it engendered, understandings of the ReSPECT form and associated processes (e.g., review of the form), and views about emergency care and treatment planning (see Supplementary Files 1 and 2). Semi-structured interviews were conducted with patients, the relatives of patients who lacked capacity, and pairs of patients and relatives (where the patient had asked for their relative to be present). Three researchers with a social science background conducted the interviews (JH, a social and policy scientist; CJH, a research psychologist; KE, a medical anthropologist). The interviews were audio recorded and professionally transcribed.

### Analysis

We approached the data from a critical realist perspective, which frames participants’ experiences as a means to understanding social processes [13]. We used thematic analysis using a critical approach, considering language as something that creates rather than reflects reality and interrogating patterns of meaning emerging from the data [14]. We used an inductive approach to theme identification [15].

KE familiarised herself with all the data, coded the interviews using both semantic and latent codes, wrote summaries that captured the key features of each interview, both descriptive and analytic, and made reflexive notes. Then, she abstracted the initial codes into higher-order codes and recoded the transcripts. Based on this second stage of coding, KE developed candidate themes. JH and CJH between them read and identified candidate themes from eight interviews. The three researchers met to discuss all candidate themes, identify potential disagreements, and reach consensus on how to develop the themes further. The final set of themes was critically reviewed and agreed upon by all co-authors.

## Descriptive findings

Fifteen potential participants sent an expression of interest; of these, two did not lead to an interview (one was uncontactable and the other could not recall having a ReSPECT form). Thirteen interviews took place (six with patients, four with relatives, and three with participant pairs of patient and relative). The patients in focus were aged 53 to 93 years (median 83 years) and included eight women and five men. Of the 13 interviews, nine took place over the phone and four in-person. Nine interviews were conducted with participants from geographic area 1, one with a participant from geographic area 2, and three with participants from geographic area 3.

In the 13 interviews, nine participants or participant pairs said they had a ReSPECT discussion with a healthcare professional, while four said they did not. Of the nine participants who had a ReSPECT discussion, eight had the discussion in person (five at home, three at the GP surgery) and one over the phone; five recalled discussing ReSPECT with a GP, three with a nurse, and one with a physiotherapist. In addition, participants mentioned hospital consultants, palliative care nurses, and social care professionals who had been involved in other aspects of the ReSPECT process – from introducing the idea of ReSPECT to completing the form. The four participants who did not have a discussion included two relatives whose parents had the ReSPECT conversation in their care homes and who said that the ReSPECT conversation took place without their knowledge. The other two participants were a patient who said the hospital doctors who completed her ReSPECT form did not discuss it with her, and a participant pair who did not recall having a discussion. These participants could not explain how and precisely when their ReSPECT forms had been completed.

Two participants had direct experience of the ReSPECT recommendations being put into practice. Relative 9 described how an ambulance driver did not transport her mother to the hospital when he read her preferences, not to be admitted to hospital, recorded on her ReSPECT form. Patient 12 said she had confidence in the ReSPECT form because ‘*it worked for my husband*’, implying his recorded wishes had been followed. For the remaining participants, however, ideas about how ReSPECT would work in practice were anchored in perceptions and understandings of the process rather than in actual events.

## Thematic findings

### ReSPECT records a patient’s wishes, but is entangled in wider relationships

The participants described ReSPECT as a record of a patient’s wishes, to be used in emergencies and in cases where the patient could not communicate. However, while participants asserted their individual decision-making as key to ReSPECT, they also described ReSPECT as involving relational concerns. This focus on relationality was evident in participants’ repeated assertions that ReSPECT was helpful to healthcare professionals and families. Participants conceptualised healthcare professionals as benefiting from ReSPECT because it would help their decision-making process and reassure them they were acting in accordance with patients’ wishes. This was sometimes related to how healthcare professionals framed ReSPECT, as described by Relative 1, who said her mother’s GP explained the form would help her mother’s carers. In another example, Relative 9 described how, at the beginning of the Covid-19 pandemic, she had been *‘bombarded’* repeatedly by her mother’s GP with questions about end-of-life planning, which she found ‘*a bit annoying*’ and related to *‘pressure on the doctors’*; once the ReSPECT form had been completed, these questions came to a stop.

Participants asserted that ReSPECT was helpful to families on a number of levels. Some participants conveyed that, in recording their wishes, ReSPECT provided relatives with clarity, enabling them to convey these wishes to healthcare professionals. When asked by the researcher what ReSPECT meant to her, Patient 5 replied:

*Well, it means that (…) if I’m taken ill at any time by a copy being left where, in easy access, it helps the paramedics or doctors who are treating me, and it, it, because I can give a copy to my daughter, she knows exactly what has been wrong with me and what my wishes are if anything happens to me. And I think it puts her mind at rest, puts my mind at rest because I know what I want will be done.*

In other cases, ReSPECT provided a platform for family negotiation, and participants described how they approached family members who disagreed with their preference not to have CPR – either by avoiding discussion of ReSPECT with them, or by explaining their wishes until they agreed. For example, Patient 4, whose children were opposed to her preference for palliative care, explained her decision to them by contextualising it within medical evidence about her condition:

*I explained to them, after speaking to the consultant and he pointing out, that the [condition] cannot be stopped. All it can be is perhaps halted for a little with tablets which may, you might not be suitable candidate for. So there’d be a lot of tests before I’d be allowed to have the medication. Along with the medication would be extreme side effects. And I, I concluded that that, for me, just to give me, say, three months more of life, now, I’d rather live my life like this with no interference, no constant treks to hospitals for tests.*

Notably, no participant reversed a decision due to relatives’ disagreement. Patient 11 said he would have changed his decision had his children objected, yet asserted that *‘[a]t the end of the day, it’s, it’s, it’s my decision, really, if I want to be, if I don’t want to be brought back, which I don’t’*.

Most participants spoke about caring for their families’ emotional wellbeing as part of the ReSPECT process. Several participants described the ReSPECT form as helpful to their families, even more so than to themselves, suggesting that the form’s main benefit was in reducing their children’s future decision-making burden; indeed, Relative 1 said she would complete a ReSPECT form for herself for this reason alone: *‘I wouldn’t like my children to have gone through what I did with my mum, and you know, to be able to have something like that in place, it, you know, it does make, make it easier‘*. Another type of relational concern revolved around reducing relatives’ caregiving responsibilities, with some participants (e.g., Patient 8) explaining that having witnessed how caring for ailing relatives influenced family dynamics, they did not wish for their own lives to be prolonged. In another example, while Patient 3 was clear about his wishes for palliative care, he said he would consider temporarily changing these if it meant he could have a few additional weeks to ensure his spouse’s financial wellbeing. What united these varied relational concerns was the understanding that while patients could and should assert their own wishes, they were networked individuals, entangled in kin relations that could not be separated from how their decisions were made and communicated.

### Healthcare professionals’ framings of ReSPECT influence patients’ and relatives’ experiences

Healthcare professionals played a key role in facilitating the ReSPECT process for patients and their relatives. In particular, patients and relatives were affected by the quality of the ReSPECT conversation. Participants who had positive experiences described open discussions in which the healthcare professional asked them questions, listened to their wishes and preferences, and provided explanations and reassurance. For example, having received a terminal diagnosis, Patient 4 was clear about her wishes for palliative care, which she immediately communicated to her consultant. The next day, a team of district nurses and a physiotherapist arrived at her home with the ReSPECT form. She was encouraged to communicate her wishes, was told she could change her mind and have her new wishes recorded, and was instructed to place the form where paramedics could easily locate it. Likewise, Patient 5 described how a nurse sensitively facilitated her ReSPECT conversation, and although she forgot the specific questions and answers exchanged, she remembered the emotions the conversation engendered and this carried forward in her accurate understanding of and positive attitude towards ReSPECT. Moreover, good rapport with the facilitating healthcare professional could transform the ReSPECT process experience, as explained by Relative 9, who began the ReSPECT conversation feeling uneasy but grew more confident in the process and gained clarity about her decision-making, crediting the GP for providing explanations and advice: ‘*Mum’s GP was very helpful in saying, “Well, if, if you have this treatment it will have these consequences on her,” and that was useful’*.

Whereas most participants had taken part in a ReSPECT conversation, some participants said they did not have a ReSPECT conversation at all. Relative 10 and Relative 6 said they had not been included in ReSPECT conversations in which they said they should have participated, as the lasting power of attorney (LPA) holders for their parents, who they felt lacked capacity. Notably, these conversations took place in care homes, as a standard procedure for new residents, while Covid restrictions were in place. Because they had not been included in these conversations, both Relative 10 and Relative 6 described feeling negatively about the ReSPECT process with regard to their parents, despite understanding the purpose of the form and ultimately agreeing with the recommendations that had been recorded. Relative 6 questioned the process, saying ‘*whilst I agree with the outcome I, I’m rather worried about the way it was gained*’. She suggested that, because the ReSPECT form had been completed during the Covid-19 pandemic, when her father was in isolation, it was done in a moment of vulnerability: ‘*he must have been feeling pretty lonely and low at that point, so perhaps he did feel really miserable and thought, “Yes, I’m going to sign it”’.* Relative 10 used the words ‘*annoying*’ and ‘*upsetting*’ to describe how she felt when, at the start of the pandemic, she was contacted by a doctor who told her that her mother, who was in hospital, would not be admitted to ICU if she contracted Covid (‘*that really, really sticks in my mind as a really bad thing*’). Later, when she discovered her mother had been issued a ReSPECT form with a ‘not for CPR’ recommendation in her care home, she felt ‘*bother[ed]*’ by what seemed to her one of several ‘*snap decisions*’ healthcare professionals had made about her mother. Although her mother had been ill for a long time, Relative 10 at first felt negatively about the recommendation against CPR attempts. However, she said she later changed her mind and agreed with this as her mother’s condition deteriorated:

*I suppose I just didn’t like the idea that, that that was slapped on, because maybe, depending on what had happened, you know, the, it, you know, I just thought it was a very, sort of, final thing to have, you know, put on [the not for CPR recommendation].*

How healthcare professionals framed certain aspects of ReSPECT within the conversation also influenced patients’ and relatives’ understandings of the ReSPECT review process. When prompted by the researcher to reflect on whether and when the ReSPECT form should be reviewed, most participants suggested it would be appropriate if there were a change in the person’s condition. However, many were unaware the ReSPECT form could be reviewed. Like other participants, Relative 1 first learned that ReSPECT could be reviewed when the researcher asked about it. She said, *‘I’ve never questioned it if I’m honest with you, I just thought, “That was it,” I weren’t aware that it had to be reviewed’*. In contrast, where healthcare professionals had framed the ReSPECT form as dynamic, participants actively considered the possibility of review should their wishes change over time. Patient 4 said, *‘they said it wasn’t written in stone, I could change my mind at any time. I haven’t at the moment changed it’*, linking this to some doubts she had about her recorded preferences following opposition from her children. Along similar lines, Patient 8 described her GP as saying *‘oh, we’ll see you again in a year and, you know, we’ll look at it again and see if it’s still the same’*, explaining that *‘people could change their mind, couldn’t they?’*.

Healthcare professionals’ framings of ReSPECT influenced not only how participants understood and felt about ReSPECT, but also how they felt about their own role in the process. Whereas many of the participating patients described feeling positively about their own preferences and decision-making, the interview with Relative 1 captured how a lack of careful framing by a GP could leave a relative feeling burdened with responsibility. ReSPECT entered this participant’s life as an administrative hurdle – she needed to have a form in place for her mother to have carers come into her home. The participant then booked a GP appointment, where the form was framed as reflecting her wishes rather than her mother’s, and where she felt compelled to provide immediate responses:

*…it threw me a bit because I, I didn’t know there was such a thing as, as a ReSPECT Form and having you know, a really close relationship with my mum and then all of a sudden I’ve found myself having to make these difficult personal decisions without consulting my mum.*

Although Relative 1 did not regret what had been decided, she regretted not having had more time to consult with her mother and other relatives, and thereby feeling alone in the decision-making process. Describing how she felt about this process, she said, ‘*it was a big responsibility to make the decisions*’. Her lingering sense of being burdened with responsibility came across toward the end of the interview, when she expressed frustration about not having been prepared for the ReSPECT process by her mother’s GP:

*I [have] always been named on my mum’s GP records. So for instance [if] she needed a medication review or anything else, it, the doctors always rang me to discuss things with me, so why did he never say, “Oh, by the way, do you know the, that this form exists?”*

### Patients and relatives perceive ReSPECT as a do-not-resuscitate or end-of-life form

Across the interviews, participants spoke of ReSPECT as a do-not-resuscitate or end-of-life form. This perception reflected the timings and contexts of the ReSPECT conversations the participants had experienced. In most cases, the ReSPECT conversation was initiated by a healthcare professional following a life-limiting diagnosis or transition to a care home. In some cases, the ReSPECT conversation was initiated by the patient, with the express purpose of avoiding CPR or other critical interventions. For example, Patient 3 was introduced to the ReSPECT form by his palliative care nurse, in response to concerns he raised about potential future treatments:

*And I spoke about some of my concerns and particularly started to talk about end-of-life care. And at that point, [Name], my palliative care nurse, said, “There is a thing called a ReSPECT form where you can lay out some of your wishes in these areas and I’ve got one here with me if you want to have a look at getting one filled out now.”*

While some participants described nuanced recommendations, such as admission to hospital in case of an injury or reversible cause, they overwhelmingly perceived the form as concerned with preparing for later stages of illness and death. Most participants framed this positively, saying they wished to avoid suffering, maintain their quality of life, and die peacefully, and that the form empowered them in that regard. For example, both Patient 7 and Patient 8, who actively sought to have a not-for-CPR recommendation recorded, said the wishes documented on their ReSPECT forms reflected their religious beliefs, which called for respecting natural death.

In a few cases, participants expressed discomfort with the ReSPECT process and form stemming from their framing of ReSPECT as an end-of-life document. Patient 12 first encountered the ReSPECT process when her husband was diagnosed with a life-limiting condition. When she was given her own life-limiting diagnosis, she was reluctant to engage in a ReSPECT discussion with her GP, feeling that it signalled an approaching terminal stage of illness:

*At first, you know, when she was saying about filling it in, I said, “Oh, I don’t feel I’ve got to that stage yet.” And she said, “Well, the thing is, if you fill it in before you get to that stage, it takes some of the pain out of it, as it were, the emotional pain.“*

In two other cases, where relatives had described negative experiences of the ReSPECT process related to how the conversation had been conducted (see Theme 2), they said that it conferred an unhelpful, even harmful, label, connoting that the patient was either giving up on life (Relative 6) or that healthcare professionals were giving up on the patient (Relative 10).

### Patients’ and relatives’ relationships with the ReSPECT form as a material object vary widely

Some participants invoked the reassuring power of having their wishes recorded in writing on the ReSPECT form. For example, Patient 12 repeated several times that her wishes had been recorded *‘in black and white’*, saying she was confident they would be carried out. When asked by the researchers what impact their ReSPECT form would have on their care, most participants said they expected their wishes will be followed. Some expressed more nuanced views, saying they were aware that medical decisions may be made before the ReSPECT form was seen by healthcare professionals (Patient 3) or that these decisions may be made regardless of the form (Relative 6).

However, despite the importance participants assigned to the form as a record of one’s wishes, the material place it held in their daily lives varied widely. In some cases, the ReSPECT form, as intended, was held by the patients themselves, with some participants treating it as a precious material object. Patient 8 provided the clearest example: she spoke of storing her ReSPECT form in a box in her refrigerator, placing a sticker on her front door to direct paramedics to the refrigerator box, and carrying a copy of the form in her purse. Yet, several participants did not know where their completed ReSPECT form was stored. In some interviews, participants searched through piles of documents, attempting to locate the ReSPECT form, at times confusing it with other forms (including the study invitation letter). Not having the form readily available also revealed a lack of awareness about how the ReSPECT form would be accessed in an emergency. Patient and Relative 13, sifting through documents, expressed the mistaken belief that once the form was completed, it would be accessible to medical teams through the patient’s medical records. Likewise, Relative 2 assumed incorrectly that if paramedics were to attend their home, the team would have access to the form on the patient’s medical records: *‘the paramedics can get access through the laptops or whatever you call them so they would know by that wouldn’t they, if the doctors have got it on record’*. This misunderstanding was likely linked to the way in which the form had been completed: Patient and Relative 2 did not recall having a ReSPECT conversation with a healthcare professional and were not aware the form should be patient-held. Along similar lines, Patient and Relative 11, whose interview began with looking for the ReSPECT form, did not know that the form had to be kept at home within easy reach, or that it was patient-held – indeed, Patient 11 apologised to the researcher for not sending the form back to the GP.

## Discussion

In this community-based interview study with patients and relatives who had a ReSPECT form completed, we found that while the participants understood ReSPECT as a record of a patient’s wishes, their experiences of ReSPECT were entangled in wider relationships. Although participants asserted their decision-making autonomy, they also spoke about caring for their families’ emotional wellbeing as part of the ReSPECT process – either through negotiating family disagreement about their treatment preferences, or through recording treatment preferences that would reduce family members’ future caregiving and decision-making responsibilities. Healthcare professionals’ framings of the ReSPECT process and form had a profound influence on how participants understood, experienced, and engaged with ReSPECT. In cases where healthcare professionals provided clear information about and preparation for ReSPECT, engaged in open discussion about patient preferences, provided empathetic explanations, and raised the possibility of the ReSPECT form being reviewed and changed, participants spoke of ReSPECT as a positive process that provided them with a sense of autonomy and peace of mind. However, in cases where healthcare professionals did not prepare participants for the conversation, did not provide clear information and explanations, or did not engage in a ReSPECT conversation, participants’ experiences ranged from confusion about the form and how it would be used to lingering feelings of worry, upset, or being burdened with responsibility, stemming from how the ReSPECT process had been conducted. The influence of healthcare professionals’ framings was also evident in the participants’ understandings of ReSPECT as an end-of-life or do-not-resuscitate form, which reflected the timings and contexts in which healthcare professionals had brought ReSPECT to the participants’ attention. The specific context of the Covid-19 pandemic also appears to have influenced the quality of communication with relatives.

Our finding that experiences of ReSPECT were relational corresponds with international scholarship on advance care planning (ACP), which has highlighted the centrality of interpersonal and socio-emotional concerns in patients’ ACP decision-making [16]. A systematic review found that cancer patients tended to prioritise their family’s wellbeing when making ACP-related decisions, despite ACP’s focus on facilitating patients’ autonomous decision-making [16]. Likewise, a study with haemodialysis patients who underwent ACP processes found that participants focused on alleviating their family’s burden [17]. This has been conceptualised as “relational autonomy” [18]. According to Oshana, the concept of relational autonomy recognises that people are social actors whose decision-making is constructed, understood and enacted in dialogue with culture, community, and society [19]. Moreover, as Stoljar and Mackenzie argue, because differences in structures and contexts lead to disparities in people’s decision-making possibilities, relational autonomy underscores how individual decision-making about healthcare cannot be detached from the social values, relationships, and inequalities that surround it [20]. In the ACP context, relational autonomy has been understood through patients’ accounts of folding their family’s possible future trajectories into their own end-of-life decision-making [18]. Critiques of ACP have posited that relational decision-making fundamentally unsettles the concept of “choice” on which ACP is premised [21]. However, our findings suggest a more nuanced interpretation, as relational concerns did not obviate choice; indeed, participants described advocating for their treatment preferences when faced with family disagreement, rather than backtracking on these preferences, thereby conveying a co-existence of personal choice and social agency.

The influence that healthcare professionals’ framings had on participants’ experiences of ReSPECT echoes broader scholarship on the role of healthcare professionals in facilitating ACP discussions. A review of reviews found that patients and relatives identified the quality of their relationships with the facilitating healthcare professionals as a key factor in ACP experiences [22]. Good rapport with healthcare professionals was also highlighted in a study of ACP facilitated by general practice nurses, where patients cited the nurses’ “compassionate and caring” approach as important to the discussion [23]. In a recent US-based study, patients identified good ACP discussions as premised on trust and rapport between healthcare professional and patient, and as those where clear information and communication about the process and its related records were provided [24]. As we found in our earlier study on hospital-based ReSPECT conversations, doctors are acutely aware that good rapport and trust are crucial to a positive experience of the ReSPECT process, and this influences their decision making on when to initiate ReSPECT conversations and with whom [7]. While the ReSPECT form is structured to facilitate consistently meaningful conversations between healthcare professionals, patients, and their relatives, our findings underscore the importance of context and quality of communication for patient and relative experiences, and, by extension, for the realisation of ReSPECT’s aims.

That some participants reported not being aware of a ReSPECT form completion for their relative is of concern. It is of note that these examples were in the context of the Covid-19 pandemic. The impact of the pandemic on emergency care treatment planning conversations was the subject of a Care Quality Commission review which found that in some cases people were not always aware that a DNACPR recommendation had been made. The report emphasised the need for healthcare providers to ensure that people and/or their representatives are included in conversations about DNACPR decisions, and emergency care treatment planning more broadly, in a way that meets people’s needs and protects their human rights [25]. Our findings reiterate the importance of healthcare professionals’ maintaining a person-centred approach to ReSPECT conversations regardless of context and setting.

The study is limited by constraints on participant recruitment. Given the study’s design, compared to potential participants who did not express interest in the study, those who chose to participate may have been more comfortable with discussing ECTP with a researcher; as such, their experiences and views might not be representative of patients and relatives who feel uncomfortable with ECTP. However, to assure the validity of our findings, we relied on researcher reflexivity and peer discussions within the team [26].

## Conclusion

Patients valued the opportunity to express their wishes and conceptualised ReSPECT as a process of caring for themselves as well as their family members’ future emotional wellbeing. Working with a healthcare professional to co-develop a ReSPECT record of treatment preferences and recommendations could be an empowering experience, providing patients and relatives with peace of mind. In ReSPECT conversations where this was achieved, healthcare professionals clearly explained the ReSPECT process and form to patients and their relatives, allocated sufficient time for an open discussion of patients’ preferences, and provided clear and empathetic explanations of treatment recommendations.

### Electronic supplementary material

Below is the link to the electronic supplementary material.

Supplementary Material 1

Supplementary Material 2

## Data Availability

Although data in this qualitative study were pseudonymised, it is possible that with access to raw data individuals might be identifiable. The data are not suitable for sharing beyond what is contained within the manuscript. Further information regarding can be obtained from the corresponding author.
