# Supplementary material for: Patient and relative experiences of the ReSPECT process in the community: an interview-based study
Source: BMC Prim Care. 2024 Apr 17;25:115. doi: 10.1186/s12875-024-02283-x (PMC11022317; doi:10.1186/s12875-024-02283-x)
Supplement: Supplementary file 2 — Supplementary Material 2 [file 12875_2024_2283_MOESM2_ESM.docx]

**ReSPECT in Primary Care**

**Evaluating the Recommended Summary Plan for Emergency Care and Treatment** **(ReSPECT) in Primary Care**

**Draft Topic Guide for Relative Interviews**

***Interview 1***

The interview topic guide will be further developed and refined during the study in collaboration with our PPI advisory group.

Topics for discussion:

1. Recollection of any ReSPECT conversation

Draft prompt question

- Do you/does your relative have a copy of your ReSPECT form?

1. Understanding about the ReSPECT process and form

Draft prompt question:

- What does the ReSPECT form mean to you?

1. Description of the ReSPECT process from when it was first considered or mentioned, through to completion of the form, and any updating of the form

Draft prompt questions:

- Can you describe the conversation with your relative’s doctor/nurse about the ReSPECT form?
- What did they tell you and your relative about the form?
- What kind of questions did they ask your relative? you?
- Who else was involved in the conversation?
- What happened to the form after it was filled in?
- Do you know if your relative’s doctor has referred to the form when deciding about their treatment when they are ill?

1. Views on the ReSPECT process

Draft prompt questions:

- When do you think people should have conversations about these kind of issues?
- Who do you think should keep the ReSPECT form?
- When do you think the ReSPECT form should be reviewed
- Who should initiate this review
- When and how it should the recommendations be acted on?

1. Thoughts and feelings that arose involvement in the ReSPECT process

Prompt questions:

- Did the process change how you thought about your relative, their health/illness, and the prospect of their death
- Do you think the ReSPECT process resulted in a change to the care and treatments your relative received,
- Has it made you think differently about their treatments
- Do you think going through the ReSPECT process with your relative has made you trust your doctors and nurses more? Less?
- How confident are you that the recommendations on the form will be acted on
- Can you think of how the ReSPECT process or form can be improved

1. Involvement in other advance care planning conversations

Draft prompt questions

- Have you had any other conversations about making plans for your relative’s future care and treatment?

1. Whether COVID-19 has had any impact on thinking about ReSPECT or advance care planning in general

Draft prompt questions

- Has COVID made you think about these kind of things more than before? If so in what way?

1. Any other comments
